# The association between active participation in a sports club, physical activity and social network on the development of lung cancer in smokers: a case-control study

**DOI:** 10.1186/1756-0500-5-2

**Published:** 2012-01-04

**Authors:** Anna Schmidt, Julia Jung, Nicole Ernstmann, Elke Driller, Melanie Neumann, Andrea Staratschek-Jox, Christian Schneider, Jürgen Wolf, Holger Pfaff

**Affiliations:** 1 Institute for Medical Sociology, Health Services Research and Rehabilitation Science (IMVR), Faculty of Human Science and Faculty of Medicine, University of Cologne, Eupener Strasse 129, Cologne 50933, Germany; 2 Gerhard Kienle Institute for Medical Theory, Integrative and Anthroposophic Medicine; Integrated Curriculum for Anthroposophic Medicine (ICURAM), Medical Department of the Private University of Witten/Herdecke, Gerhard-Kienle-Weg 4, Herdecke 58313, Germany; 3 LIMES (Life and Medical Sciences Bonn), Genomics and Immunoregulation, University of Bonn, Karlrobert-Kreiten Strasse 13, Bonn 53115, Germany; 4 Department III for Internal Medicine, University Hospital of Cologne, Kerpener Strasse. 62, Cologne 50937, Germany; 5 First Department of Internal Medicine, Molecular Tumour Biology and Tumour Immunology & Centre for Integrated Oncology (CIO), University Hospital of Cologne, Kerpener Strasse 62, Cologne 50937, Germany

**Keywords:** Social network, Sports club, Physical activity, Lung cancer, Smokers, Germany

## Abstract

**Background:**

This study analyses the effect of active participation in a sports club, physical activity and social networks on the development of lung cancer in patients who smoke. Our hypothesis is that study participants who lack social networks and do not actively participate in a sports club are at a greater risk for lung cancer than those who do.

**Methods:**

Data for the study were taken from the **Co**logne **Smo**king **S**tudy (**CoSmoS**), a retrospective case-control study examining potential psychosocial risk factors for the development of lung cancer. Our sample consisted of n = 158 participants who had suffered lung cancer (diagnosis in the patient document) and n = 144 control group participants. Both groups had a history of smoking.

Data on social networks were collected by asking participants whether they participated in a sports club and about the number of friends and relatives in their social environment. In addition, sociodemographic data (gender, age, education, marital status, residence and religion), physical activity and data on pack years (the cumulative number of cigarettes smoked by an individual, calculated by multiplying the number of cigarettes smoked per day by the number of years the person has smoked divided by 20) were collected to control for potential confounders. Logistic regression was used for the statistical analysis.

**Results:**

The results reveal that participants who are physically active are at a lower risk of lung cancer than those who are not (adjusted OR = 0.53*; CI = 0.29-0.97). Older age and lower education seem also to be risk factors for the development of lung cancer. The extent of smoking, furthermore, measured by pack years is statistically significant. Active participation in a sports club, number of friends and relatives had no statistically significant influence on the development of the cancer.

**Conclusions:**

The results of the study suggest that there is a lower risk for physically active participants to develop lung cancer. In the study sample, physical activity seemed to have a greater protective effect than participation in a sports club or social network of friends and relatives. Further studies have to investigate in more detail physical activity and other club participations.

## Background

According to a report by the World Health Organisation (WHO), cancers are the third-leading cause of death worldwide [1]. Lung cancer, one of the most common types of cancer, is the leading cause of death in the western world and, in Germany, accounts for approximately 26% of all cancer deaths among men and 12% of all cancer deaths among women [2]. Smoking is known to be the main risk factor for the onset of this type of cancer [3,4]. Approximately 1.3 billion people worldwide--that is, nearly 1 billion men and 250 million women--currently smoke cigarettes or other products [5]. Killing nearly 4.2 million people each year, smoking is the world's greatest cause of death [6-8]. Although most lung cancer patients are smokers, only approximately 10-15% of all smokers get the disease [9]. This suggests that individual genetic or psychosocial factors may enhance or inhibit the noxious effects of smoking on the disease's development [10]. In light of the demographic changes in our society and the apparent curative limitations of therapeutic medicine, increased research in the area of preventive medicine seems more important than ever. There is little evidence that psychosocial factors like an intact social network has a positive effect on health and could have a preventive effect on the development of cancer [11].

### Social network, active participation in a sports club and physical activity

The association between social network and health was originally described by Durkheim (1951), who reported "that the lack of social networks predicted mortality from almost every case of death" [12]. Other empirical studies have shown that having a satisfying and diverse web of personal relationships, or 'social networks', has both a positive effect on mental well-being and a protective effect on physical health [13,14]. The association between psychosocial factors and the onset of disease (ischemic heart disease, cancer or stroke) has primarily been examined using very vague indicators (marital status, participation in a sports club, number of close friends) [13,15,16]. After many years of empirical research, probably one of the strongest findings of social epidemiology is that certain psychosocial factors - such as a combination of social isolation and a lack of social network - contribute to an increased risk of disease, especially in stressful situations [15,17].

Already in the year 1979 Berkman and Syme [18] conducted research in the area of social network. They found out, that using a self-developed index "people with social ties and relationships had lower mortality rates than people without such ties." Additionally, they found that the "more intimate ties of marriage and contact with friends and relatives were stronger predictors than were the ties of church and group membership." The 'Group membership' is one of the four sources (number of social ties and relatives, church affiliations) of the Social Network Index. According to Welin et al. [19], several prospective studies have been conducted linking poor social networks to increased mortality during follow-up, indicating that a poor social network is an important factor contributing to disease causation. Östergren and colleagues [20] stated that the impact of psychosocial resources, such as social network, on mortality and morbidity has gained wide recognition and that social networks and social support have been claimed to buffer the influence of stressors on an individual or even play a principal role in health promotion. Kroenke and colleagues [21] reported that socially isolated women had a higher risk of mortality after diagnosis of breast cancer. Based on the results of these studies, it is believed that decreased social support leads to an increase in the risk of mortality. Social network could take place in a sports club. Physical activity seems to have a positive health benefit and plays accordingly an important role in lung cancer prevention [22-24]. For instance, Sinner et al. [25] reported in their study that physical activity is a potentially protective factor against lung cancer. The results suggest that physical activity could reduce the risk of lung cancer in women who smoke. Physical activity is often associated with sports participation, which induces physiological changes beneficial to health.

In a search on PubMed in March 2011, using the keywords (MeSH terms) 'smoke, lung cancer, social support, social environment, health, illness, psychosocial support, social network and physical activity', we found several studies investigating the association between social network (in terms of friends or relatives) [15], physical activity [25] and different diseases (e.g. heart diseases [26] or cancer [13]). However, there are no studies carried out so far that exclusively investigated active participation in sports club and the development of lung cancer by patients with a history of smoking.

### Aim of the study

Our explorative study aims to determine whether there is an association between active participation in a sports club and social network on the development of lung cancer in people with a history of smoking when controlling for socioeconomic variables (age, gender, education, marital status, residence, religion) and other confounder variables like pack years and physical activity. Using the design of the **Co**logne **Smo**king **S**tudy (**CoSmoS**), we were able to examine the hypothesis that smokers and ex-smokers who participate in sports clubs and have a social network are at a lower risk of lung cancer.

## Methods

### Study design and participants

Data was collected from CoSmoS, a multicentre case-control study examining genetic and psychosocial factors potentially leading to a higher risk for smokers of suffering a myocardial infarction, developing lung cancer and/or becoming addicted to nicotine. Approval for the study was obtained from the Ethics Committee of the University Hospital of Cologne (UHC). All data from the participants underlie the legal requirements from the federal data security and the discretion.

Two case-study groups (acute myocardial infarction and/or a history of myocardial infarction patients and lung cancer patients) and one hospital-based control group were recruited for the study. The lung cancer patients were recruited at the UHC and Chest Clinic Merheim. The control patients were selected from the Orthopaedics and Dermatology departments from the UHC. In order to be included, participants in all three groups had to be of European descent, reside in or around the city of Cologne and be born between 1930 and 1970. The main phase of the study lasted two years (for more details on the study design, see [27]).

Of the n = 524 participants included in CoSmoS, 458 (87.4%) were smokers or ex-smokers and 66 (12.6%) were non-smokers. Smokers are people who currently smoke or have smoked at least over half a year. Non-smokers are people who have never smoked.

**Figure 1:**
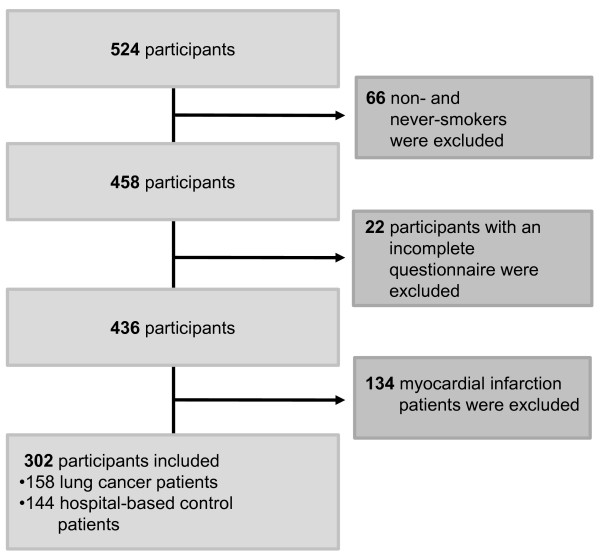
**Flow chart of the sampling procedure**.

Participants were 180 lung cancer patients and 170 myocardial infarction patients. In CoSmoS there were 174 control group patients, who had not been diagnosed with either condition and did not have an admission diagnosis of cancer or nicotine-related disease. By recruiting patients in three different control clinics, the issue of selection bias was to a large extent avoided. The control group served as the comparison group for both case-study groups. Potential participants were approached by a study nurse on the wards of the different departments. Patients who met the inclusion criteria gave written consent for participation and were surveyed in hospital through face-to-face interviews, with each interview lasting an average of 45-60 min. For the present analysis, only data from participants with lung cancer and from the control group were used. Both groups had a history of smoking (see Figure 1).

### Measures

For the present study, we have limited ourselves to an analysis of participants' friends and relatives, physical activity and their degree of active participation in sports clubs.

Information on the participants' social environment was obtained by asking two questions adapted from the Berkman-Syme Index [18]. The first question asked was the following: "During the year when you were first diagnosed with lung cancer (case-study group) or your current disease or condition (control group), how many friends/relatives (apart from you own children) did you have that you felt close to and that you could talk openly to about personal issues? The interviewer recorded these two numbers, which were then dichotomised for analysis by median split at a value of 3 into 'no/few' and 'many' (see Data Analysis). In the second question, participants were asked to recall how often they had participated in a sports club during the year when they were first diagnosed with lung cancer (case-study group) or their current disease or condition (control group). Response options were 'frequently', 'sometimes' and 'never'. The 'frequently' and 'sometimes' responses were combined into one category for the analysis.

Sociodemographic and other data (gender, age, education, religion, marital status, residence and pack years) taken from the comprehensive questionnaire of CoSmoS were included in the analysis as additional control and moderator variables. Data on gender was obtained by asking participants whether they were 'male' or 'female'. The age variable was assessed by asking patients when they were born and then categorising them into ten-year-span age groups (1 = 35-44; 2 = 45-54; 3 = 55-64; 4 = 65-76) for the analysis.

When asked about the highest level of education they had achieved, participants could respond with: (1) 'I did not complete a vocational education programme and I am not currently receiving any vocational education or training'; (2) 'I completed vocational training (*apprenticeship*)'; (3) 'I completed my education at an academically-oriented vocational school (*specialised vocational school*, *commercial college*)'; (4) 'I completed my education at a trade school, guild school, technical college for technicians, or specialised vocational college'; (5) 'I graduated from a technical university or university of applied sciences'; (6) 'I have a university degree'. The education responses were then combined into three categories for the statistical analysis: 0 = no vocational/university education (1), 1 = lower vocational education (2-4), and 2 = higher vocational/university education (5-6).

Religion was assessed by asking patients whether they practised any faith (e.g. Christianity, Sikhism, Islam, Judaism) or not. For analytical purposes, the religious categories were dichotomised into 'religious' and 'not religious'.

For marital status, patients could respond with: (1) ‚'married and living with my spouse', (2) 'married but not living with my spouse', (3) 'not married', (4) 'divorced' or (5) 'widowed'. These responses were then dichotomised into 'married and living together' (1) and 'not married and/or not living together' (2-5).

To determine place of residence, patients were asked which of the following categories best described where they were living: (1)'I live in a large city', (2) 'I live on the outskirts or in the suburbs of a large city', (3)'I live in a medium-sized or small city', (4) 'I live in a small town or village' and (5) 'I live on a farmstead or in a house in the country'. Responses were combined and dichotomised as 'city' (1-3) and 'country' (4-5).

To measure physical activity, patients were asked about their activity during the year prior to the first diagnosis of their current condition. Responses were coded as 0 (not physically active during the week) and 1 (physically active at least one hour per week).

The pack years variable, a measure of the cumulative number of cigarettes smoked by an individual, was calculated by multiplying the number of cigarettes smoked per day by the number of years the person had smoked divided by 20 [28] and categorised as none, 15 or fewer, more than 15 [29]. Pipe and cigar smoking were not examined in this study.

Due to the measure description, lung cancer patients or control patients are the dependent variable. The independent variables are gender, age, education, marital status, residence, religion, physical activity, pack years, friends, relatives and participation in a sports club.

### Data analysis

Figure 1 illustrates the sampling procedure for the statistical analysis. Lung cancer patients and control group patients were asked the same questions from the comprehensive CoSmoS survey. Participants with missing or incomplete data for social status or social network were excluded from analysis.

Stem-and-leaf plots were constructed to identify any outliers before conducting the bi- and multivariate analysis.

A two-step analysis was then conducted. First, Spearman and chi-square tests of association were performed on the study's independent variables to determine whether there was a statistically significant difference in the means of the two patient groups. Next, we tested our hypothesis using a stepwise logistic regression model because the logistic function was needed to estimate the probability that study participants belonged to one of the binary dependent variable categories coded '0' for control patients and '1' for lung cancer patients. An analysis of residuals indicated that the residuals were not normally distributed and therefore exhibited nonlinearity.

Statistical data were analysed using IBM SPSS Statistics 19.

## Results

### Descriptive findings

Our sample consisted of n = 302 participants with a history of smoking, of whom n = 158 were lung cancer patients and 144 were control group patients. The distributions are shown in Table 1.

**Table 1:** The characteristics of the CoSmoS-study sample (n = 302)

Variable	Coding	Missing values	Lung cancer patients		Control patients	
		**n**	**n**	**%**	**n**	**%**

Gender	male	0	104	65.8	89	61.8

	female		54	34.2	55	38.2

Age	35-44	2	3	1.9	23	16.1
	45-54		44	28.0	31	21.7
	55-64		61	38.9	51	35.7
	65-76		49	31.2	38	26.6

Education	no vocational/university education	3	34	21.8	15	10.5
	lower vocational education		113	72.4	102	71.3
	higher vocational/university education		9	5.8	26	18.2

Marital status	married	3	48	30.6	46	32.4
	not married		109	69.4	96	67.6

Residence	city	2	36	22.8	25	17.6
	country		122	77.2	117	82.4

Religion	religious	0	36	22.8	37	25.7
	not religious		122	77.2	107	74.3

Physical activity	physically active at least one hour per week	1	44	27.8	72	50.3
	not physically active during the week		114	72.2	71	49.7

Pack years	≤ 15 pack years	10	91	59.5	109	78.4
	> 15 pack years		62	40.5	30	21.6

Friends	many	0	72	46.6	72	50.0
	none/few		86	54.4	72	50.0

Relatives	many	0	72	45.6	79	54.9
	none/few		86	54.4	65	45.1

Participation in a sports club	yes	0	19	12.0	35	24.3
	no		139	88.0	109	75.7

The results of the stem-and-leaf plots revealed high outliers for both number of friends (mean = 6.2; maximum outlier = 150) and number of relatives (mean = 5.4; maximum outlier = 50). Two separate regression analyses were conducted, one with and one without the detected outliers (median split at 3) [30]. No significant differences were found without the outliers present. The outliers were excluded for the present regression analysis.

### Bivariate analysis

The results of the Spearman and chi-square tests yielded no significant correlations between the independent variables (results not shown here). Similarly, none of the variables under investigation demonstrated intercorrelations > 0.80, which indicated that there was no multicollinearity [31].

### Multivariate analysis

The results of the stepwise logistic regression are shown in Table 2.

**Table 2:** The results of a stepwise logistic regression (CoSmoS n = 302)

	unadjusted Model 1				adjusted Model 2			
Independent variable	Beta	SE	OR	CI	Beta	SE	OR	CI
Gender (male^#^)	-0.35	0.29	0.23	0.399-1.24	-0.42	0.29	0.66	0.371-1.17
Age								
35-44^#^								
45-55	2.63	0.70	13.90***	3.55-54.39	2.96	0.71	14.75***	3.70-58.78
55-64	2.26	0.68	9.62***	2.53-36.55	2.27	0.69	9.66***	2.49-37.44
65-76	2.09	0.69	8.09**	2.08-31.39	2.06	0.70	7.86**	1.99-30.99
Education								
no vocational/university education^#^								
lower vocational education	-0.63	0.39	0.53	0.25-1.15	-0.53	0.40	0.59	0.27-1.28
higher vocational/university education	-1.57	0.55	0.21**	0.07-0.61	-1.54	0.55	0.21*	0.07-0.63
Marital status (not married^#^)	0.21	0.29	1.24	0.70-2.19	0.28	0.30	1.32	0.74-2.36
Residence (country^#^)	-0.51	0.35	0.60	0.30-1.18	-0.50	0.35	0.61	0.30-1.21
Religion (not religious^#^)	0.30	0.31	1.35	0.73-2.47	0.41	0.32	1.51	0.81-2.81
Physical activity (not physical active^#^)	-0.86	0.27	0.42**	0.25-0.72	-0.63	0.31	0.53*	0.29-0.97
Pack years (≤ 15^#^)	0.03	0.01	1.03*	1.01-1.06	0.03	0.01	1.03*	1.01-1.06

Friends (none/few^#^)					-0.16	0.27	0.85	0.50-1.46
Relatives (none/few^#^)					-0.38	0.28	0.69	0.40-1.18
Participation in a sports club (no^#^)					-0.51	0.38	0.60	0.28-1.26

Cox and Snell pseudo-R2	.19				.20			
Nagelkerke pseudo-R2	.25				.27			
McFadden pseudo-R2	.15				.16			

Note: # = reference response, Beta = regression coefficient, CI = confidence interval, Odds = odds ratio, SE = standard error, *p *≤ 0.05*, *p *≤ 0.01**, *p *≤ 0.001***

In the following, we report the results of Model 2. (For the results of Model 1, please see Table 2.) In this model, physical activity (adjusted OR = 0.53*) has a positive benefit against the development of lung cancer. The results also indicated that participants aged 45-55 had significantly higher risk of lung cancer (adjusted OR = 14.75***) than those aged 35-40. For participants in the 55-64 and 65-76 age groups, the risk was even greater (adjusted OR = 9.66***; adjusted OR = 7.86**). Participants with a higher level of education (*higher vocational/university education*) had the lowest risk of developing lung cancer (adjusted OR = 0.21**). This risk became higher as the level of education decreased. In Model 2, the likelihood of developing lung cancer was greater in those with a > 15 pack-year smoking history (adjusted OR = 1.03*). No other independent variables had a statistically significant influence on the onset of lung cancer.

In Model 2 the Nagelkerke pseudo-R^2 ^was 27% (for the other coefficients, see Table 2). The specificity of the second model was 60%; the sensitivity was 76%.

## Discussion

### Main findings

Our research question was to investigate whether active participation in a sports club and social network by relatives and friends has a protective effect against the development of lung cancer. The research on psychosocial factors like social network and the onset of lung cancer in smokers is insufficient. Using the data of CoSmoS we were able to determine that partly very ill participants with a history of smoking who were physically active are at a lower lung cancer risk. In our study we found no association between active participation in a sports club and social network of friends and relatives on the development of lung cancer. The Sinner [25] research team also found that physical activity might reduce the risk of lung cancer in women who are current or former smokers. According to our results, physical activity is of positive benefit to the health of the participants. The consideration that regular participation in a sports club, together with the opportunity this provides to meet others with similar interests, is an appropriate resource for preventing lung cancer (community effect) could not be confirmed in this study population. In our study it seems that physical activity is a higher protective factor than active participation in a sports club. For this study sample social network with like-minded people is not an important factor for preventing lung cancer. Another study found that the interaction effect--talking with like-minded people in a sports club--has a positive effect on health [32]. The study conducted by Lames and Kolb [33] found out, that physical activity in general and in particular in sports club has a positive effect to health.

In our sample we also analysed participation in other clubs like occupational or church organisations. Even this shows no association between club participation and the development of lung cancer (results not shown here).

The degree of integration in a social environment, measured by the number of friends and relatives, does not have a significant effect on the onset of lung cancer. These results differ from the findings of other studies reporting an association between psychosocial variables, such as social network (number of friend and relatives), and the onset of disease [10,20]. Contrary to our results though, Berkman and Syme [18] attributed greater significance to the impact of contact with friends than to the ties of church and group membership.

Other sociodemographic characteristics, such as increasing age and lack of a vocational or university education, constitute disease risk factors. As in other studies, the participants in our study who had a vocational or university education were found to have a lower lung cancer risk than those without a higher level of education. In addition, participants' risk of lung cancer was demonstrated to increase with age [34]. This result shows that older people have a higher risk of cancer not least because of the dimension of smoking history [35]. Participants with a heavy smoking history (> 15 pack years) were at a greater lung cancer risk. Numerous empirical studies have also found a correlation between the amount smoked and the onset of lung cancer [36]. Our study did not detect any significant effects of place of residence, religion, marital status or gender on the development of lung cancer. Numerous other studies, however, offered differing results [37], such as those which found that unmarried men died earlier from cancer than married men [17,19].

### Limitations of the study

Due to the retrospective design of CoSmoS, there may be memory distortions in the participants' responses. The retrospective nature of the study also makes it difficult to draw cause-and-effect conclusions [10]. Unlike the other studies we mentioned, our study surveyed severely ill participants; face-to-face interviews had to be conducted in hospital and were not anonymous. The presence of another individual at these interviews, such as a patient or visitor, may have been enough to distort the results [38]. Social desirability, which involves the systematic distortion of responses in a certain direction, may have distorted the marginal distributions of the participants' responses and must be considered when looking at the study's results [39].

Research studies on issues concerning social support often report having used the Berkman-Syme Index. However, when looking at the actual application of the index in these studies, it becomes apparent that different studies frequently use different self-developed procedures for data collection and analysis. This makes it difficult to draw direct comparisons between studies, which should be taken into consideration.

Moreover, not everyone likes to participate in clubs or groups; being happy and healthy does not necessarily require active participation in a sports club. In our study, only a limited number of participants with a history of smoking participated in sports clubs, which suggests that smokers may have lesser inclination to participate in sports clubs. In CoSmoS we could not investigate whether physical activity was practised in leisure-time or in a sports club. Due to the study's sample size, the number of independent variables examined for their association with lung cancer had to be limited. An excess of parameters and associated overfitting of the data would have led to unstable regression coefficient estimates [40].

Moreover, it is possible for a pre-existing, undetected tumour to have an impact on certain personality traits. If this was the case in our sample, it may have distorted patients' statements regarding social network and, consequently, the study's results [41].

### Future research

The findings of previous studies and this study point to some areas in need of further investigation. In CoSmoS all participants were very ill. The protective factors have already failed. Future research should include prospective studies with larger, non-inpatient, healthy samples of participants with a history of smoking from various professional fields.

Future studies should investigate more precisely the benefit of sports clubs (contact with other sports club participants) besides the physical activity. Further research could investigate where participants do physical activity, whether in their leisure-time or in a sports club. Future studies should analyse more precise how often participants were physically active. The individual needs of the participants, as regards social network, should also be taken into consideration. In addition, investigation into other forms of club participation (e.g. occupational or church organisations) seems important.

Given the multifactorial nature of cancer, attempts at reducing the risk of developing this disease should focus on the patient as a whole rather than on individual factors. In doing so, it is of utmost importance that as much psychosocial and sociodemographic information is taken into consideration as possible [16]. Therefore, it seems worthwhile to conduct prospective studies measuring health status and support from active participation at different points in time, in order to determine whether changes in the social environment have a direct impact on the onset of cancer.

In addition to the examination of psychosocial factors, a further research question, in relation to data from CoSmoS, is possible, analysing which genetic traits account for the onset of lung cancer in certain smokers, while others are not affected by the disease.

## Conclusion

For the high-risk group of ill smokers physical activity seems to be a possible factor that protects against lung cancer. For the participants in our study the physical activity is more important than the active participation in a sports club and the network of relatives and friends. A physician could suggest to patients physical activity. Interventions targeted at psychosocial risk factors can also have many positive effects by enabling people to modify their unhealthy behaviour and to reduce the negative consequences of stress [42]. Moreover, the medical system could play a major role in providing patients with the physical activity needed to develop healthy behaviours and improve their compliance. Patients with risk factors, such as too little physical activity, may benefit from interventions tailored to their specific needs, both in terms of information and emotional support. The study results showed that the extent of smoking has a negative effect on the development of lung cancer. For the risk group 'current smokers', smoking reduction or cessation should also be considered for inclusion in therapeutic regimens [43]. The risk of lung cancer decreases shortly after quitting smoking [44].

## Competing interests

The authors declare that they have no competing interests.

## Authors' contributions

AS was the lead author of the manuscript. HP, JW, CS, AS-J and MN participated in the design of the study. AS and JJ worked together with two other research assistants to collect the data from the face-to-face interviews. AS, JJ and ED directed the statistical analyses. NE prepared the analyses and tables. All authors read and approved the final manuscript.
